# Differences in School Performance Between Only Children and Non-only Children: Evidence From China

**DOI:** 10.3389/fpsyg.2021.608704

**Published:** 2022-01-13

**Authors:** Chaochao Jia, Zhaoxi Yang, Tao Xin, Youfa Li, Yehui Wang, Tao Yang

**Affiliations:** ^1^The Collaborative Innovation Center of Assessment for Basic Education Quality, Beijing Normal University, Beijing, China; ^2^Shanghai Minhang Education Evaluation and Research Center, Shanghai, China

**Keywords:** only child, mathematics achievement, physical fitness, school well-being, propensity score

## Abstract

This study aimed to investigate the features of only child status related to physical health, mathematics achievement, and school feelings and expectations from a different perspective. A representative sample of 91,619 Grade 4 students with an average age of 10.4 ± 0.7, among which 28,631 were only children, were assessed. We used propensity score matching (PSM) and the average treatment effects on the treatment to analyze data. The treatment was the only child of a family. The results indicated that only children have better academic achievement and school feelings (only for urban only child girls), while non-only children have better physical status and anaerobic fitness (AF). In addition, gender and rural vs. urban areas differences were also explored. The adverse situation for rural only boys is emphasized for families, researchers, and governments to focus on. Some suggestions are given under the Two- and Three-Child Policy.

## Introduction

The special group named as “The Only child” has attracted researchers’ interest for a long time. Since the 19th century, Western researchers have paid attention to the only child. An only child is defined as a child who does not have any siblings (Burke, 1956; Cai et al., 2012), and has been firstly viewed as “a problem child” for hundreds of years throughout the world (Goodenough and Leahy, 1927; Su, 1994). Later, Norman Fenton criticized that kind of view, as well as the aspects of methods and contents in his representative “The Only child” (Fenton, 1928), and since then the arguments about the only child have not stopped. In the 1980s, Toni Falbo, the Department of Educational Psychology at the University of Texas, published a series of research reports and journals (Falbo and Polit, 1986; Polit and Falbo, 1987), as well as the book “The Single-child family” (Falbo, 1985) involved in the overall assessment of 115 pieces of research of the only child in 100 years, which was reviewed by many researchers all over the world (Dunham, 1985; Guerney, 1985; Maruyama, 1985; Chafetz, 1986; Koch-Hattem, 1986), and explored a wide range of impact conditions. This book indicated some advantages of only children compared with non-only children and eliminated the labels once attached to the only child, such as selfishness, self-centeredness, immaturity, and low social ability. However, the discussion on this topic has never stopped, and the stereotype that only child is a problem child still runs deep.

In China, the Family Planning Policy was written into the Constitution in 1982 (National People’s Congress [NPC], 1982) and lasted until the end of 2015 (Feng et al., 2016). As a result of more than three decades of policy implementation (Feng et al., 2016), now China has the largest population of only children in the world (Falbo and Poston, 1993), both in quantity and proportion. We are eager to find out whether the status of “only child” affects the school performance.

Recently, a lot of empirical research concerning the only child has emerged. However, several researchers only cared for one part of the students’ development such as cognitive achievement (Gaynor and Runco, 1992; Jiao et al., 1996; Liu et al., 2010, 2019; Duncan et al., 2011; Zheng et al., 2014; Chu et al., 2015; Zhang and Shen, 2018), personality or mental health/emotion outcome (Burke, 1956; Polit and Falbo, 1987; Duan, 1998; Ma and Xu, 1999; Li and Zhang, 2001; Liu et al., 2003; Zhong et al., 2005; Yuan et al., 2013; Wu, 2014), the physical status (Hesketh et al., 2007; Hunsberger et al., 2012; Li et al., 2017; Li and Liu, 2019), and behavior (Zhang, 2002). Some researchers considered the two different kinds of students’ performance, mainly on academic achievement (cognitive achievement) and personality characteristics (emotional outcome) (Goodenough and Leahy, 1927; Bao et al., 1989; Lam, 1992; Liu et al., 2017). Even a very few studies examined three or more parts of the outcomes of Only child (Falbo and Polit, 1986; Falbo et al., 1989; Falbo and Poston, 1993), and the studies were conducted years ago.

Peoples’ full development includes rich content such as intelligence, physique, and psychology. It was said in “The Republic” (Plato, translated by Guo and Zhang, 1986), that an all-round development of moral, physical, intellectual, and aesthetic education was needed for a person’s overall development. Focusing on only one or two aspects is not enough to cover students’ school performance. It is necessary to examine the recent situation of the only child’s all-round development of intelligence, physique, and psychology status, especially after the long period of the Only Child Policy in China. To learn about an all-round influence of Only child status, we versatilely explored whether the only child status affects mathematics achievement (MA), physical fitness, school feeling, and expectations of the school. The development of academic achievement is not only the main aspect of school-related educational achievement but also an important dimension of child development (Zhang et al., 2012). Academic achievement was highly appreciated by parents, teachers, principals, and educational departments, and was thought to be the most significant outcome of schooling. According to economic theory, the process of child academic development is the process of human capital accumulation. Micro studies show that the better children perform in primary and secondary school, the more likely they are to earn higher incomes upon entering the labor market (Lazear and Oyer, 2004).

Many studies have investigated the association of only child status and mathematic achievement, mostly indicating that only child status has advantages (Zheng et al., 2014; Liu et al., 2017). Few studies indicated that non-only children have better academic achievement than only children (Huang and Wen, 2008). Still, others found that there was no significant difference between the only child and non-only child (Lam, 1992). These inconsistent results may be due to the unrepresentative samples or the unreliable methods.

Schools not only nurture academic achievement but also promote students’ health and wellbeing (Jourdan et al., 2008). Health is also crucial for one’s development. Besides, students’ physique is fundamental to school physical education and other activities. Previous studies have indicated a positive association between physical education and MA, and PE curriculum implementation may benefit students’ academic achievement (Ericsson and Karlsson, 2014; Hansen et al., 2014; Donnelly et al., 2016; Mullender-Wijnsma et al., 2016; Wang et al., 2019). Thus, PE education is not only important for your physical fitness but also important for your academic success.

The relationships between Only child and physical fitness are also observed. Some studies have pointed out that only children have better physical condition (Wolley et al., 1990), but other studies have indicated that only children also have a higher rate of overweight or obesity (Kobzova et al., 2004; Hunsberger et al., 2012; Ochiai et al., 2012; Li and Liu, 2019). And the longer a child has been an only child, the higher the risk of being overweight (Hesketh et al., 2007; Hunsberger et al., 2012).

In recent years, there is a growing concern about health. Also, studies on PE are increasing. However, most of the studies in western countries are divided and are mainly about physical health. There are only a limited number of studies available on anaerobic fitness (AF) and cardiorespiratory fitness (CF), which are also important physical education outcomes. More research is needed on AF and CF in China.

Happiness is the goal of life. School wellbeing (SWB), which is students’ subjective happiness in school environment experience (Tian and Liu, 2007), should also be the goal of school life. Schoorl (1994) mentioned that SWB referred to students’ perception of school life satisfaction and feeling at ease in the school. SWB is the result of students’ interaction with people, things, and environment. It refers to students’ evaluation and experience of school life based on their own criteria, which is composed of school satisfaction, positive emotional experience, and negative emotional experience (Tian and Liu, 2007).

School is an important place for learning and social interaction among children. During the daytime, students spend about 40 h per week in school, 8 h a day from Monday to Friday, accounting for nearly half of their waking time. A large amount of schooling time determines that students’ psychological development is bound to be affected by school life. A positive school climate is associated not only with higher academic achievement but also with better self-reported student health, wellbeing, and health behaviors (Cohen et al., 2009; Jia et al., 2009) and lower perceived stress (Torsheim and Wold, 2001). Better feelings and expectations have been proven to lead to higher academic achievement (Huebner and McCullough, 2000; Cheng, 2010; Ru and Wu, 2010; Yu, 2017), while excessive expectations from parents will result in poorer learning achievement (Du, 2018).

Parents’ expectation is defined as the plan or design for their children’s future based on parents’ experience, knowledge, and thought (Buck, 1991), while students’ expectation is based on the information that students mastered or analyzed from their own perspective. Expectation contains the expectation and assumption of children’s academic performance and future development, including educational achievement (Yang and Wu, 2000; Wu, 2003; Song et al., 2007).

Learning expectations have a direct impact on students’ mental health (Wang, 2006; Zhang and Huang, 2014) and learning outcomes (Zhao, 2001; Tang, 2014). Parental expectation was positively related to their children’s mental health (Zhang and Huang, 2014), while students’ learning expectation has a significant prediction on students’ learning outcomes (Tang, 2014). However, parents’ excessive high expectations will cause children psychological pressure, which may lead to children’s self-abasement, and frustrated self-confidence (Chang, 1981; Li J., 2003; Sui, 2004).

The special status of only children may influence their own school feelings and learning expectations as well as those of their parents. Wang (2020) indicated that the SWB of an only child is significantly higher than that of a non-only child. Zhang and Huang (2014) found that there was no significant difference between only child’s parental expectancy and non-only child’s parental expectancy, while Li (2007) found that whether a child is an only child or not has a significant impact on parents’ expectations. However, there is no consistent result on the influence of only child status on school feelings and learning expectations.

Some variables other than only child status may also affect the results of students’ school performance, such as provincial Gross Domestic Product (GDP), region, school location, students’ age, gender, ethnicity, boarding status, migrant status, kindergarten education, and family structure and socioeconomic status (SES). Higher academic achievement is related to higher SES (Coleman, 1966; Deng and Treiman, 1997; Zhou et al., 1998; DeGarmo et al., 1999; Li C., 2003; Li, 2006, 2016; Liu, 2008; Sun et al., 2009; Wu, 2009, 2013; Duncan et al., 2011; Hansen et al., 2014). The migrant status also affects students’ academic performance, and the students left behind in rural have poor performance (Luo, 2014). Family SES also has an important influence on family educational expectation, and higher family’s expectation for children’s education is related to a higher SES (Liu et al., 2014; Ren and Dong, 2017). The Han ethnic parents have higher expectations for children’s education than ethnic minorities (Liu et al., 2014; Ren and Dong, 2017). The regions where the family resides also affect children’s academic performance (Xie, 2017). Urban families have higher learning expectations than rural families (Liu et al., 2014). Rural children who have siblings and boarding at home have better academic achievement than their classmates boarding in school (Nie et al., 2016). Children’s gender and their parents’ education level will influence the possibility of overweight or obesity of children (Hesketh et al., 2007; Hunsberger et al., 2012). The influence of genders on SWB is not consistent, some indicated that girls have higher SWB (Epstein and McPartland, 1976; Okun et al., 1990; Karatzias et al., 2002; Wan, 2017; Wang, 2020), while others found that there is no difference in school satisfaction between boys and girls (Shmotkin, 1990; Huebner et al., 2001). The positive emotion of students from rich families was significantly higher than that of students from poor families (Wang, 2020). Thus, all these covariates should be adjusted in predicting propensity scores and outcomes.

The family education investment varies with the number of children. The Resource Dilution Theory may explain the differences in family resources divided by children with a different number of siblings, gender, and birth order, which will affect children’s educational opportunities (Marjoribanks, 1991; Downey, 1995). The theory of resource dilution assumes that family resources are limited, so as the number of children in a family increases, each child shares less resources in the family (Downey, 1995). While making education investment choices, families with the maximum number of children have certain gender and age preferences (Gong and Zhong, 2006; Ren et al., 2009), but families with the minimum number of children are more willing to make a high investment (Gong and Zhong, 2006). Among large families in East Asia, the elder girls fared the worst (Parish and Willis, 1993). Parents may devote excessive resources to investing the younger boys at the expense of the elder girls’ resources (Chu et al., 2007; Xie et al., 2018). In rural families with a maximum number of children, parents invest less in education and pay less attention to children’s academic performance than those with a minimum number of children (Ren et al., 2009). As a result, it would be expected that the only children would have excessive resources and opportunities, and thus have better school performance than those of non-only children. This study’s research question is whether the status of “Only child” affects school performance. If we detected some adverse situations, suggestions and interventions would be initiated earlier.

## Materials and Methods

In China, the Collaborative Innovation Center of Assessment for Basic Education Quality (CICA-BEQ) launched the Chinese National Assessment of Education Quality (CNAEQ) with authorization from the Ministry of Education of the People’s Republic of China (MOE of PRC) in 2015. The CNAEQ is China’s nationally representative education quality assessment. The assessment is conducted across a 3-year period with two disciplines every year (Wu et al., 2019). Participants were sampled by probability proportionate to size across 323 counties in China.

Our study used the propensity score matching (PSM) method to explore whether only child status affects MA, physical fitness, and school feelings and learning expectations, based on national data of the CNAEQ. The main research questions studied include the following. (1) What are the characteristics of only children and non-only children, and how do they differ? (2) Does only child status affect the MA, physical fitness, school feeling, and expectations of school? (3) Do the differences vary between subgroups, including boys vs. girls and urban vs. rural areas.

### Data and Sample

The data used in our study were collected from Chinese National Assessment of Education Quality [CNAEQ] (2015). Chinese National Assessment of Education Quality [CNAEQ] (2015) was carried out on June 18, 2015. Thirty-one provinces (or municipalities, hereinafter referred to as provinces) and one Corp, Xinjiang Production and Construction Corp, which is a provincial unit in China with a separate education and teaching system, participated in national assessments representing Mainland China. To be representative at the national level, a three-stage stratification cluster sampling design with systematic probability proportional to size (PPS) technique was employed (Zhang and Tang, 2017). First, counties in provinces were selected according to their GDP and educational development levels in the first stage. Second, 12 primary schools were selected from each county based on their location, schooling quality, and school size. Third, 30 students were randomly selected within each school. If the total number of schools in a district was less than the demand, then the number of schools in that district would be the number of school samples or the number of students. For detailed information about the assessment design, organization, and procedure of the assessment, see Wang et al. (2019) and Wu et al. (2019). In addition, quality control and incident management are considered thoroughly in advance and are well implemented during the whole test (Wu et al., 2019). The final sample used in this study consisted of 91,619 Grade 4 students (with an average age of 10.4 ± 0.7 years), 28,631 students were only children and 62,988 students were non-only children. Among them, 21,445 students were excluded for missing either control or independent variables.

The assessment took place on the same day for every province. CICA-BEQ trained a professional team around the country to take on the test organizing during the assessment. The mathematics test took place in the morning as well as the questionnaire, and the physical test took place in the afternoon. It took 80 min to finish the mathematics test and 60 min for the questionnaire including students’ demographic characteristics, family characteristics, and some questions about school learning. Grade 4 students were chosen because they are more cognitively developed than their younger counterparts, and therefore their reading comprehension and written expression are more likely to be reliable (Wu et al., 2019). Grade 4 is considered a critical period for children’s learning and habit development (Zhao, 2017).

### Measures

#### Mathematics Achievement

Mathematics achievement was assessed using students’ paper–pencil test. The mathematics examination measured mathematical knowledge and capability, including five main abilities such as arithmetic ability, reasoning ability, statistical ability, spatial-imagination ability, and problem-solving ability. The designation of tests was based on the compulsory education mathematics curriculum standards (Ministry of Education of the People’s Republic of China [MOE], 2012).

All the items were designed for the test on purpose. To ensure test quality, all items had undergone two pilot tests and at least three rounds of expert review and modification before all were used in the national examination. The experts who participated in the mathematics items review include mathematicians, mathematics educators, mathematics teaching and research staff, and mathematics teachers, so are the tests for physical and questionnaire. We administrated each pilot test in three counties, from east China, middle China, to west China. In each county, we selected three school types according to the teaching quality. More than one thousand students in each county participated in each pilot tests. After each pilot test, we examined the items’ difficulty, differentiation, and the test’s length, validity, and reliability.

At last, six parallel tests were used, which contain 12 multiple-choice items and 6–9 construct response items, whose internal consistency was 0.85–0.88. Each student should finish one test. The Rasch model and concurrent calibration were used to link the scores of the different test booklets to an identical scale provided by Conquest 1.1 (Wu et al., 1998). The item difficulty ranged from -2.84 to 3.56 logits. A new scale was generated, which ranges from 229 to 768 with a mean of 500 and a SD of 100 (Chinese National Assessment of Education Quality [CNAEQ], 2015; Wang et al., 2019).

#### Physical Fitness

##### Body Mass Index

We collected students’ height and weight in the field assessment using standard measuring equipment recommended by the organization.

Body mass index (BMI) was calculated as weight (kg) divided by the square of height (m) (Fredriks et al., 2000; Rolland-Cachera and FTECO Group, 2011).

(1) $$\mathrm{BMI}=\frac{\text{Weight}\,\left(\mathrm{kg}\right)}{{\text{Height}}^{2}\,\left(m^{2}\right)}$$

##### Anaerobic Fitness

Speed is assessed for AF. We used a 50-m sprint as the predictor of speed. In the field test, the time students took to finish the 50-m sprint was collected by recorders with unified training. The more time one takes, the worse AF has. The overflow values are dealt with according to the “Sports Monitoring Indicators Data Processing Instructions,” exceeding the range from 6.5 to 16 s.

##### Cardiorespiratory Fitness

The Progressive Aerobic Cardiovascular Endurance Run (PACER) is a widely used and recommended field test with established validation and reliability to assess CF (Mahar et al., 2006). It is easily administered to students and easily scored. We used the 15-m PACER in the field test, and the more laps one runs, the better CF one has. Scores were capped at 44 laps for boys and 35 laps for girls in Grade 4 (Wu et al., 2019).

#### School Feeling and Expectations

##### School Wellbeing

School wellbeing was measured by six items (e.g., “I feel like staying at school” and “I learn a lot from school”). This instrument employed a 4-point Likert-type scale response format, ranging from 1 (strongly disagree) to 4 (strongly agree). Responses to the scale indicated the extent of agreement with each item. All items were from the student questionnaire. The mean score of each item ranges from 1 to 4, with higher scores suggesting happier school feelings. The sixth item (I wish I did not have to go to school) was reverse coded. The means of the six items were calculated, and the samples who answered more than three items were taken into account. The scale’s internal consistency was acceptable (α = 0.67).

##### Learning Expectation by Students

Learning expectation by students (LES) was measured by one item, asking students to answer the following question: “What is your expectation for your learning achievement?” All items were from the student questionnaire, which employed the 4-point Likert-type scale response as well. Higher scores suggested higher learning expectations by students.

##### Learning Expectation by Students’ Parents

Learning expectation by students’ parents (LESP) was also measured by one item, asking students to answer the following question: “What is your parents’ expectation for your learning achievement?” All items were also from the student questionnaire, with a 4-point Likert-type scale response. Higher scores suggested higher learning expectations by parents as perceived by students.

### Analytic Strategy

#### Propensity Score Matching

Regarding only children’s academic achievement and physical condition, the previous findings are not consistent (Falbo and Polit, 1986; Polit and Falbo, 1987; Falbo et al., 1989; Falbo and Poston, 1993). As the status of being an only child or a non-only child is unchangeable, it cannot be treated as a control variable in the experiment. Therefore, it calls for a reasonable method with more robust properties to study the differences between only children and non-only children. The PSM method is one of these methods, which is defined as the conditional probability of receiving “treatment” (Rosenbaum and Rubin, 1983; Dehejia and Wahba, 2002; Imbens, 2004; Liu et al., 2010). It is often used to estimate the effects of experimental treatment in clinical medicine, epidemiology, economics, and other fields, where randomized experimental treatment is not available, and minimize the effect of the confounding variables on the results (Rosenbaum and Rubin, 1983; Xin and Li, 2009; Liang, 2010; Yuan et al., 2013).

Propensity score matching generally consists of two steps: estimating propensity scores and matching participants based on propensity scores. The propensity score is defined as the conditional probability of receiving “treatment” (Rosenbaum and Rubin, 1983; Dehejia and Wahba, 2002; Imbens, 2004; Liu et al., 2010).

When establishing the propensity score model, it is necessary to take into account all the observable covariates related to the outcome variables. The more the selected covariables, the closer it is to a randomized trial (Brookhart et al., 2006). For this reason, when selecting covariates in this study, variables related to students’ school performance including academic, physical, and emotion results were all included. The covariates included in our study are provincial GDP, region, school location, students’ age, gender, ethnicity, boarding status, migrant status, kindergarten education, and family structure and SES.

The covariates are measured, calculated, and coded as follows: GDP was derived from the National Bureau of Statistics of China [NBSC] (2017). The region referred to in which part of China (eastern/middle/western) the school is located, the migrant status indicated living accompaniment (migrant in urban/left behind in rural/ordinary), and both of them were recoded into two dummy variables (Judd et al., 2017). Age was calculated in both years and months while students took the assessment. For SES, a principal component analysis was conducted based on the three indicators, including the educational level of parents, the occupational status of parents, and home material possessions, and the first principal component was used as the index of SES (Organization for Economic Co-operation and Development [OECD], 2014). Others are dichotomic variables, of which school location was divided into two categories (urban or rural), gender (boy or girl), ethnicity (Han or others), boarding status (home or school), kindergarten education (experienced or not), and family structure (intact or broken).

In this study, the treatment was defined to be the only child in one’s family. To estimate propensity scores, we fitted a logistic model with demographic and family characteristics as the independent variables and only child status as the dependent variable. The logistic model returned a fitted probability that a student was only child, and this estimated probability was the propensity score.

Based on the propensity scores, we used two different matching algorithms and estimated the average treatment effects on the treated (ATT). The matching algorithms pairs treatment and comparison units with similar propensity scores. We are ultimately interested in estimating the ATT of only child status on academic performance, physical fitness, and school feelings and expectations.

First, we used nearest neighbor matching (NNM). This matching is done with replacements to ensure that each treatment unit is matched to the nearest comparison unit in the propensity score and thus can maximize the reduction in selection bias (Imbens, 2004). Unmatched comparison units were removed. After matching, ATT was estimated by the following model:

(2) $$A\,T\,T=\frac{1}{N^{T}}\,\sum_{i\in T}y_{i}^{T}-\frac{1}{N^{C}}\,\sum_{j\in C}y_{i}^{C}$$

where $y_{i}^{T}$ denotes the math score of student *i* in the treatment group (*T*), and $y_{i}^{C}$ is the “nearest neighbor” *j* in the control group (*C*) that is matched to *i*; *N^T^* and *N^C^* denote the number of treated units and control units, respectively.

We also use stratification matching (SM) for a robustness check. SM incorporates a tradeoff between the quality and quantity of matches different from NNM (Becker and Ichino, 2002). We used five layers in estimating the ATT as it was indicated that when the number of layers was five in the propensity value stratification model, approximately 90% of the deviation in confounding factors could be eliminated (Cochran, 1968). The ATT was calculated as follows:

(3) $$A\,T\,T=\sum_{q=1}^{Q}\left(\frac{\sum_{i\in I\,\left(q\right)}y_{i}^{T}}{N_{q}^{T}}-\frac{\sum_{i\in I\,\left(q\right)}y_{i}^{C}}{N_{q}^{C}}\right)\,\frac{\sum_{i\in I\,\left(q\right)}D_{i}}{\sum_{\forall i}D_{i}}$$

where observations are divided by blocks *Q* defined over intervals of the propensity score; in each block, *q* treated unites and control unites have balanced covariates; ATT in each block *q* is then weighted to generate the overall ATT with the block weighting function $\frac{\sum_{i\in I\,\left(q\right)}D_{i}}{\sum_{\forall i}D_{i}}$.

Nearest neighbor matching and SM were conducted using the *matchit* packages in R (Ho et al., 2011). ATT was estimated using the *zelig* package in R (Choirat et al., 2020). The present study examined the outcomes of only child status with MA, PE outcomes, school feelings, and expectations. We also examined how such associations may vary by gender and school location.

#### Effect Size

The effect size (ES) reflects the difference in SD between the mean values of two distributions, which represents the actual difference between two populations despite the sample size. We use Cohen’s *d* to represent the ES, which was calculated as follows:

(4) $$d=\left(\bar{y_{1}}-\bar{y_{2}}\right)/\sigma_{p\,o\,o\,l\,e\,d}$$

(5) $$\sigma_{p\,o\,o\,l\,e\,d}={\left[\frac{\left(n_{1}-1\right)\,s_{1}^{2}+\left(n_{2}-1\right)\,s_{2}^{2}}{n_{1}+n_{2}}\right]}^{1/2}$$

A Cohen’s *d* of 0.2, 0.5, and 0.8 indicates a small, medium, and large ES, respectively (Cohen, 1969).

For a very large sample, small differences will be statistically significant. We calculate the ES, which is a more valid indicator, to testify the differences between only child and Non-only child.

## Results

### Descriptive Statistics on Students and Their Families

Table 1 presents the descriptive statistics on students and their families by demographic characteristics. The percentage ratio of only children in the sample is 31.3%. Male-only children (56.8%) is higher than female-only child (43.2%). There are also maximum number of children in the eastern area (42.6%) and urban area (65.8%), and more children (95.3%) in these areas had experienced kindergarten education.

**TABLE 1:** Sample characteristics.

Variable	Full sample	Only children	Non-only children
Only children (%)	31.3	–	–
**Students’ demographic characteristics**			
Age (years)	10.4	10.3	10.5
Gender (%)			
Boys	51.6	56.8	49.2
Girls	48.4	43.2	50.8
Ethnicity (%)			
Han	86.8	89.9	85.4
Else	13.2	10.1	14.6
Region (%)			
East	36.3	42.6	33.3
Middle	29.1	26.9	30.0
West	34.6	30.5	36.7
School location (%)			
Urban	48.5	65.8	40.6
Rural	51.5	34.2	59.4
Boarding condition (%)			
Home	88.9	92.9	87.1
School	11.1	7.1	12.9
Migrant status (%)			
Migrant in Urban	4.5	4.0	4.7
Left behind in rural	8.4	5.3	9.8
Ordinary students	87.1	90.7	85.5
Kindergarten education (%)			
Experienced	93.4	95.3	92.6
Non-experienced	6.6	4.7	7.4
**Family characteristics**			
Family structure (%)			
Intact	82.7	83.8	82.2
Broken	17.3	16.2	17.8
Socio-economic status (SES)	0.03	0.42	-0.14
***N***	91619	28631	62988

**FIGURE 1:**
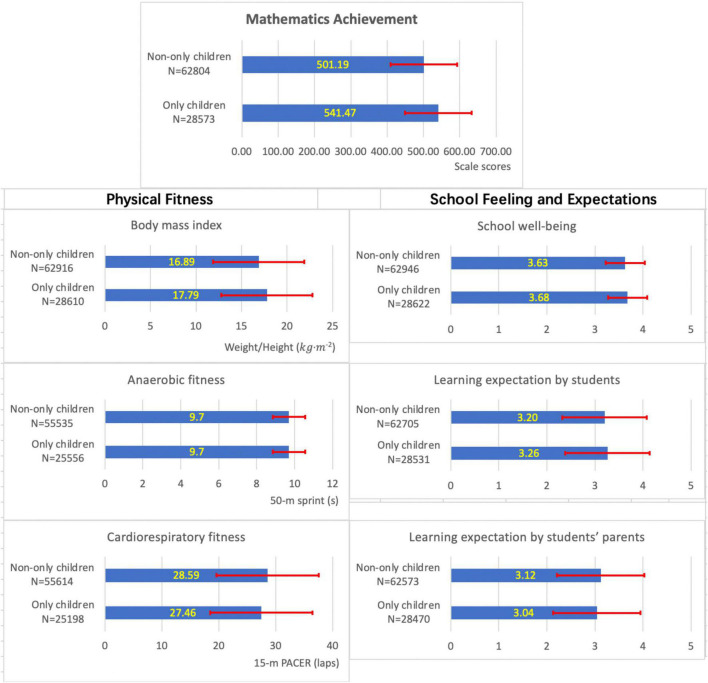
Outcomes of only children and non-only children.

Figure 1 compares the outcomes of the only children and non-only children.

### Conducting the Propensity Score Matching Steps

Table 2 shows the differences in demographic characteristics with original groupings, and the data before and after applying the two PSM estimates, NNM and SM.

**TABLE 2:** Differences in demographic characteristics before and after matching.

Variable	Sample	Only child	Non-only child	*p*-Value	Cohen’s *d*
Age	Before	10.32	10.46	<0.001	**0.20**
	NNM	10.32	10.33	0.74	0.00
	SM	10.32	10.34	0.42	0.05
Gender	Before	1.43	1.51	<0.001	**0.15**
	NNM	1.43	1.44	0.04	0.02
	SM	1.43	1.44	0.20	0.04
Ethnicity	Before	1.10	1.15	<0.001	**0.14**
	NNM	1.10	1.10	0.91	0.00
	SM	1.10	1.11	0.24	0.04
GDP	Before	57376.67	49115.42	< 0.001	**0.39**
	NNM	57376.67	55129.22	<0.001	0.10
	SM	57375.69	56378.12	0.09	0.10
Region-east	Before	0.43	0.33	<0.001	**0.19**
	NNM	0.43	0.40	<0.001	0.05
	SM	0.43	0.42	0.05	0.05
Region-middle	Before	0.27	0.30	<0.001	0.07
	NNM	0.27	0.29	<0.001	0.04
	SM	0.27	0.27	0.49	0.02
Location	Before	1.34	1.59	<0.001	**0.52**
	NNM	1.34	1.36	<0.001	0.03
	SM	1.34	1.35	0.14	0.05
Boarding condition	Before	1.93	1.87	<0.001	**0.19**
	NNM	1.93	1.92	<0.001	0.03
	SM	1.93	1.92	0.09	0.07
Migrant status-migrant	Before	0.04	0.05	<0.001	0.03
	NNM	0.04	0.04	0.03	0.02
	SM	0.04	0.04	0.29	0.05
Migrant status-rural residency	Before	0.05	0.10	<0.001	**0.17**
	NNM	0.05	0.05	0.44	0.01
	SM	0.05	0.05	0.26	0.02
Kindergarten education	Before	1.95	1.93	<0.001	**0.11**
	NNM	1.95	1.95	0.91	0.00
	SM	1.95	1.95	0.49	0.03

**Family characteristics**					

Family structure	Before	1.16	1.18	<0.001	0.04
	NNM	1.16	1.17	0.05	0.02
	SM	1.16	1.17	0.16	0.08
Socio-economic status (SES)	Before	0.42	–0.14	<0.001	**0.60**
	NNM	0.42	0.33	<0.001	0.09
	SM	0.42	0.37	0.01	0.08

*NNM, nearest neighbor matching; SM, stratification matching.*

Demographic groups had statistically significant differences before matching. Initial Cohen’s *d* of students’ age, family SES, school location, and provincial GDP turned out to be more than 0.2, which means that there were differences between the two groups. The other variables, such as gender, ethnicity, region, boarding condition, migrant status, and kindergarten education experience, have a lower but notable Cohen’s *d* value (from 0.1 to 0.2). After applying the PSM estimates, Cohen’s *d* of all variables drops to less than 0.1. The results prove that our PSM procedures are valid and effective.

### Estimation Results of “Only Child” Status

We then studied causal effect estimation results of “only child” status. In addition, we also examined how such associations may vary by gender and school location.

Tables 3.1, 3.2 show the differences between only children and non-only children using NNM and SM, respectively. The results of the two methods turned out to be similar.

**TABLE 3.1:** The differences of OC and NOC using NNM.

Outcome variables	Parameter estimates	All samples (*n* = 57262)	Boys (*n* = 32522)		Girls (*n* = 24740)	
			Urban (*n* = 20892)	Rural (*n* = 11630)	Urban (*n* = 16806)	Rural (*n* = 7934)
MA	Beta	19.48***	21.19***	17.32***	20.13***	16.67***
	*SE*	0.53	0.89	1.23	0.91	1.40
	Cohen’s *d*	0.24	0.30	0.18	0.28	0.17
BMI	Beta	0.55***	0.72***	0.61***	0.45***	0.45***
	*SE*	0.03	0.06	0.06	0.04	0.08
	Cohen’s *d*	0.13	0.14	0.14	0.12	0.09
AF	Beta	1.73***	1.68***	2.04***	1.86***	2.93***
	*SE*	0.20	0.34	0.46	0.38	0.55
	Cohen’s *d*	0.04	0.03	0.06	0.04	0.09
CF	Beta	0.10	−0.31	−0.65	1.30**	1.38*
	*SE*	0.25	0.43	0.54	0.45	0.59
	Cohen’s *d*	0.00	0.02	0.01	0.02	0.04
SWB	Beta	0.03**	−0.01	0.05	0.05***	0.00
	*SE*	0.01	0.01	0.04	0.01	0.03
	Cohen’s *d*	0.02	0.00	0.02	0.05	0.00
LES	Beta	0.03	0.03	0.16	0.01	−0.33
	*SE*	0.04	0.06	0.079	0.05	0.12
	Cohen’s *d*	0.01	0.01	0.03	0.01	0.04
LESP	Beta	−0.06	−0.06	0.03	−0.02	−0.37
	*SE*	0.04	0.08	0.11	0.08	0.12
	Cohen’s *d*	0.01	0.01	0.00	0.00	0.05

OC, only child; NOC, non-only child; NNM, nearest neighbor matching; MA, mathematics achievement; BMI, Body mass index; AF, anaerobic fitness; CF, cardiorespiratory fitness; SWB, school wellbeing; LES, learning expectation by students; LESP, learning expectation by students’ parents. *p < 0.05, **p < 0.01, ***p < 0.001.

**TABLE 3.2:** The differences of OC and NOC using SM.

Outcome variables	Parameter estimates	All samples (*n* = 91619)	Boys (*n* = 47240)		Girls (*n* = 44379)	
			Urban (*n* = 23282)	Rural (*n* = 23958)	Urban (*n* = 21115)	Rural (*n* = 23264)
MA	Beta	19.80***	20.93***	17.20***	20.40***	17.23***
	*SE*	0.44	0.87	0.81	0.87	0.76
	Cohen’s *d*	0.25	0.26	0.19	0.27	0.21
BMI	Beta	0.55***	0.68***	0.54***	0.43***	0.44***
	*SE*	0.03	0.06	0.04	0.04	0.04
	Cohen’s *d*	0.12	0.12	0.14	0.12	0.10
AF	Beta	2.13***	1.65***	2.16***	1.43***	2.72***
	*SE*	0.20	0.36	0.31	0.39	0.29
	Cohen’s *d*	0.05	0.06	0.09	0.05	0.09
CF	Beta	0.49*	–0.31	0.10	0.94*	0.89**
	*SE*	0.24	0.47	0.39	0.47	0.33
	Cohen’s *d*	0.02	0.02	0.06	0.03	0.07
SWB	Beta	0.02*	–0.01	0.07***	0.05***	–0.01
	*SE*	0.01	0.02	0.02	0.01	0.01
	Cohen’s *d*	0.03	0.03	0.06	0.08	0.05
LES	Beta	–0.02	0.02	0.20***	0.00	–0.24
	*SE*	0.03	0.05	0.06	0.05	0.06
	Cohen’s *d*	0.02	0.03	0.05	0.01	0.03
LESP	Beta	–0.07	0.01	0.00	–0.05	–0.14
	*SE*	0.04	0.08	0.07	0.08	0.06
	Cohen’s *d*	0.01	0.03	0.02	0.02	0.03

OC, only child; NOC, non-only child; SM, stratification matching; MA, mathematics achievement; BMI, Body mass index; AF, anaerobic fitness; CF, cardiorespiratory fitness; SWB, school wellbeing; LES, learning expectation by students; LESP, learning expectation by students’ parents. *p < 0.05, **p < 0.01, ***p < 0.001.

#### Mathematics Achievement

In MA, being only children will receive higher scores in all kinds of samples. The statistical differences of the value of *p* < 0.001 and Cohen’s *d* range from 0.17 to 0.30. The differences between the two groups are significant.

Non-only children who transferred to only children will gain extra 18.95–20.01 points in mathematics by using NNM, and 19.36–20.24 points by using SM in all samples. Urban non-only child boys who transferred to only child boys will gain extra 20.3–22.08 points in mathematics by NNM, and 20.6–21.8 points by SM, and rural non-only child boys who transferred to only child boys will gain extra 16.09–18.55 points by NNM, and 16.39–18.01 points by SM. Urban non-only child girls who transferred to only child girls will gain extra 19.22–21.04 points by NNM, and 19.53–21.27 points by SM, and rural non-only child girls who transferred to only child girls will gain extra 15.27–18.07 points by NNM, and 16.47–17.99 points by SM.

#### Physical Fitness

In physical fitness, both only children and non-only children have their advantages.

To be only children will have higher BMI. The statistical differences of the value of *p* < 0.001 and Cohen’s *d* range from 0.10 to 0.14. Non-only children who transferred to only children will have 0.52–0.58 points higher BMI by using NNM and SM in all samples. Urban non-only child boys who transferred to only child boys will have 0.66–0.78 points higher by NNM, and 0.62–0.74 points by SM, and rural non-only child boys who transferred to only child boys will have 0.55–0.67 points by NNM, and 0.50–0.58 points by SM. Urban non-only child girls who transferred to only girls will have 0.41–0.49 points higher by NNM, and 0.39–0.47 points by SM, and rural non-only child girls who transferred to only child girls will have 0.37–0.53 points by NNM, and 0.40–0.48 points by SM.

To be only children will need more time to finish the 50-m sprint in all samples, urban boys, rural boys, urban girls, and rural girls. However, although all statistical different values of *p* < 0.001 and Cohen’s *d* is less than 0.2, which means that the ES is small. Non-only children who transferred to only children will need 1.53–1.93 more seconds to finish the 50-m sprint by using NNM, and 1.97–2.33 more seconds by SM in all samples. Urban non-only child boys who transferred to only child boys will need 1.34–2.02 more seconds to finish the 50-m sprint by using NNM, and 1.29–2.01 more seconds by SM. Rural non-only child boys who transferred to only child boys will need 1.58–2.50 more seconds to finish the 50-m sprint by using NNM, and 1.85–2.47 more seconds by SM. Urban non-only child girls who transferred to only child girls will need 1.48–2.24 more seconds to finish the 50-m sprint by using NNM, and 1.04–1.82 more seconds by SM. Rural non-only child girls who transferred to only child girls will need 2.38–3.48 more seconds to finish the 50-m sprint by using NNM, and 2.43–3.01 more seconds by SM. The rural non-only child girls status had a bigger effect on 50-m sprint.

Cardiorespiratory fitness had little differences between only children and non-only children among all groups (*p* > 0.05). However, the non-only child status had a greater impact on girls than on boys. Non-only child girls who transferred to only child girls will have better CF. Urban non-only child girls who transferred to only child girls will run 0.85–1.75 more laps in 15-m PACER by using NNM, and 0.47–1.41 more laps by using SM, and rural non-only child girls who transferred to only child girls will run 0.79–1.95 more laps by NNM, and 0.56–1.22 more laps by SM.

#### School Feeling and Expectations

Non-only children who transferred to only children will have a better feeling at school in the total sample and urban girls, but Cohen’s *d* is less than 0.2. Non-only children who transferred to only children will improve 0.02–0.04 points by NNM, and 0.01–0.03 points by SM in all samples, and urban non-only child girls who transferred to only child girls will improve 0.04–0.06 points by NNM and SM.

There were no differences in learning expectations by students and parents (LES and LESP) (*p* > 0.05), except for rural boys. Rural non-only child boys who transferred to only child boys will have higher self-expectations of learning when using the SM method (*p* < 0.001), with a slight ES of 0.05.

## Discussion

### The Characteristics of Only Children in China

The results suggest that in China, only children are more likely to be boys and have parents with a higher SES, which is consistent with Liu et al. (2017). In addition, we found that only children are more likely to be of Han ethnicity and live in the East area and urban area, which is consistent with the research of Falbo and Poston (1993) and the effects of the One-Child Policy (Central Committee of the Communist Party of China [CPC], 1983; Bongaarts and Greenhalgh, 1985). Additionally, only children are more likely to have experienced kindergarten education, which has been a new concept up until now. It also makes sense that as the family has a higher SES and lives in more developed areas, they are certainly capable and likely to send their children to kindergarten.

### Differences Between Only Children and Non-only Children

The results of our study are derived from a representative sample from China. Also, we explored the reflections of being only children of a family in different perspectives, including academic achievement, physical fitness, school feeling, and learning expectations.

#### Mathematics Achievement

In our study, we found that to be only children will receive higher scores in mathematics. This is consistent with most previous studies (Wang and Liu, 1987; Falbo et al., 1989; Falbo and Poston, 1993; Zheng et al., 2014).

The explanation of the only child’s outperformance in achievement may lie in the aforementioned facts. First, sibship size will dilute educational resources. It has been found that sibship size significantly affects the family educational investment of migrant children, and migrant families invest more educational resources in only children (Xie et al., 2018). Moreover, the dilution of the time investment is larger than the money investment dilution. Second, only children have better parent–child communication and high involvement of parents in education. Guo and Luo (2019) found that the level of parent–child communication and educational involvement of only child parents were significantly higher than those of first non-only child parents after controlling for parents’ SES. Finally, the greater achievement of only children may also be caused due to the greater intelligence and education of their parents. Some studies have suggested that the educational attainment or SES of parents is a proxy variable for their intelligence; intelligence was not measured in the assessment and was not controlled for in the analysis. It has been found that education is inversely related to fertility (Bumpass and Westoff, 1970; Westoff and Ryder, 1977). The research conducted on a nationwide sample of urban whites has also found that women with higher IQs have fewer children than women with lower IQs (Udry, 1978). Thus, it may also lead to the outperformance of only children.

Our study revealed that all groups of “only child” status had a significant advantage in their MA. However, previous studies found that only children in an urban area had higher academic performance than non-only children in the same type of area, while there was no difference in rural areas (Bao et al., 1989; Poston and Falbo, 1990). This may partly be because our samples are more representative, or the rural and urban areas are becoming increasingly similar. China has seen the largest human migration in history (Gong et al., 2012), leading to a rapid rise in the urban population. As more rural youngsters either moved to urban areas or accepted the same living concept as citizens, villagers, townspeople, and citizens are becoming more similar. Currently, rural and urban only children all perform better than non-only children in mathematics in China.

#### Physical Fitness

Childhood obesity is considered a major issue because of its high prevalence and its severe consequences on adult health (Rolland-Cachera and FTECO Group, 2011). Our study indicates that there are increased obesity risks for being an only child in China. The data of all subgroups matched the conclusion. The result coincides with Kobzova et al. (2004) and Li and Liu (2019). Li and Liu (2019) examined only children who were born from 1976 to 2001 and pointed out that all only children aged 16–40 years had a higher BMI than non-only children; this result was also proven in our study. Li et al. (2017) found that compared with sibling sons, only child sons had a higher BMI and thus higher risks of overweight/obesity, and the result pronounced overweight/obesity risks for only child sons in urban China. Now, our results show that the association is also significant for both urban girls and rural girls, as well as rural boys now, which means a higher risk of obesity and that particular attention should be paid to all groups. Overweight and obesity have been important health threats and need to be paid more attention to by individuals, families, and society (Li et al., 2017; Li and Liu, 2019).

The CF and AF have different trends between only children and non-only children. For AF, to be an only child means that one needs more time to finish the 50-m sprint, which means that non-only children perform better in terms of speed. Only children of urban and rural boys and girls all spent more time finishing the 50-m sprint, which is the same as that of all samples. However, for CF, the differences between only children and non-only children could be ignored. These outcomes have seldomly been discussed before; thus, further research is needed to explore the results and reasons behind them.

#### School Feeling and Learning Expectation

The present study found no difference in parents’ expectations of learning between them, which is different from Hao and Feng (2002). In another study, it was revealed that the expectation of only child families in underdeveloped regions is significantly higher than that in developed regions (Liu and Liu, 2004). The discordance between our study and that of Hao and Feng may be due to the objects of comparison. After controlling the covariables, parents’ expectations of children’s learning may have no distinction. The current study also found that only child status has no influence on students’ self-expectations of learning. Only rural only child boys’ self-expectation of learning was higher than that of rural non-only child boys by using SM scores. The result shows that rural only child boys have higher expectations of learning, which may mean that rural only child boys suffer from more pressures than do non-only child boys. It was reported that influenced by son preference, migrant families invest in more educational resources for boys than for girls (Xie et al., 2018). Then, when only child boys grow up, their parents are more likely to live with grown/married only child sons (Zhou et al., 2011). This means that only child boys in rural areas have more responsibility to take care of their elder parents, and thus, this may lead to high self-expectations.

The results of the present study indicated that urban only child girls are happier at school than non-only children. A previous study also found that only child college students have higher happiness viewpoints (Li and Zhang, 2001), and only children had an advantage in their general wellbeing (Falbo and Polit, 1986). Wu (2014) found that rural only child boys were associated with lower subjective wellbeing than non-only child boys in the same areas. Our results suggest that there is no SWB difference in rural only child boys and non-only child boys by using NNM, while the SM method suggests that rural only child boys have happier school feelings than non-only child boys. This inconsistency requires further studies.

### Response to the Two- and Three-Child Policy

The results of our study are derived from a representative sample from China. Also, we explored the reflections of being the only children of a family in different perspectives, including academic achievement, physical fitness, school feeling, and learning expectations.

The status of only child may benefit children’s academic achievement. According to the Resource Dilution Theory, family resources are limited, so as the number of children in a family increases, each child shares fewer resources in the family (Downey, 1995). So, to have sufficient resources to raise more than an only child, the government should provide additional assistance for the two- and three-child families. Besides, the parents of more child family should pay the same attention to children’s learning if they want to keep the good achievement, especially for the elder sisters.

The status of only child may increase obesity risks according to our study. Individuals, families, and society should pay more attention to overweight and obesity (Li et al., 2017; Li and Liu, 2019). The Two- and Three-Child Policy may relieve this risk. The status of rural only child boys may cause higher self-expectations of learning, which may cause greater pressure as well. The tradition of living and relying on boys in parents’ old age may be the cause (Hannum, 2003). The Two- and Three-Child Policy may be good news for rural only child boys. However, parents should treat sons and daughters equally. Otherwise, the daughters in the Two- and Three-child family fared poor (Parish and Willis, 1993).

### Limitations

First, although we considered the important variables that may influence the association between only child status and the outcomes, there may be some other variables left due to our limited knowledge and the limitations of the existing assessment. Second, the aspects we compared in this study include MA, physical fitness, and school feelings and expectations. They are more comprehensive than those aspects considered in previous studies, but there are still blanks in moral and aesthetic aspects that are also important for individuals’ development. Third, the sample used in this study includes only one grade. Fourth, the subsequent development of students, which would be quite interesting, is unable to be traced as the CNAEQ is a cross-cut assessment. Finally, there are still some inconsistent results using the two methods of matching, and more studies are needed, especially referring to rural only child boys.

## Conclusion

Despite all these limitations, our study is important because it adds to the literature on Chinese evidence in the following ways.

First, the sample used in this study is representative of China and thus provides Chinese evidence.

Second, we explored the reflections of being only children of a family in different perspectives, including academic achievement, physical fitness, school feeling, and learning expectations.

Third, the findings from this study suggest that several variables influence the relationship between only child status and outcomes, including age, gender, ethnicity, region, location, boarding condition, migrant status, kindergarten education, family structure, and SES. It is meaningful to use propensity scores to examine the differences between only children and non-only children, which have seldom been used before.

Fourth, our study also indicates that both only children and non-only children have significant advantages after controlling for covariates. Only children have better academic achievement and school feelings (only for urban only child girls), while non-only children have better physical status and AF. However, overweight and obesity, as important health threats, have not been an independent study topic on the relationship between the Only Child Policy and public health in China, and thus, they call for more attention.

Fifth, despite its high achievement, the results of the SM method show that only children are also under more stress, especially only child boys in rural areas. The adverse situation is warmed for families, researchers, and governments. More studies about only children in rural areas should be conducted in the future.

## Data Availability Statement

The data analyzed in this study is subject to the following licenses/restrictions: the dataset is confidential. Requests to access these datasets should be directed to YW, yehuiwang@bnu.edu.cn.

## Ethics Statement

The studies involving human participants were reviewed and approved by the Research Committee of Beijing Normal University, as well as by the Local Government Committee. Written informed consent to participate in the study was obtained from the participants’ legal guardian/next of kin.

## Author Contributions

CJ was involved in assessment design, study design, manuscript writing, and revision. ZY was involved in data analysis and manuscript revision. TX was involved in assessment design and commented on the manuscript. YL was involved in assessment design. YW was involved in assessment design, study design, data analysis, and manuscript revision. TY was involved in assessment design, study design, and manuscript revision. All authors contributed to the article and approved the submitted version.

## Conflict of Interest

The authors declare that the research was conducted in the absence of any commercial or financial relationships that could be construed as a potential conflict of interest.

## Publisher’s Note

All claims expressed in this article are solely those of the authors and do not necessarily represent those of their affiliated organizations, or those of the publisher, the editors and the reviewers. Any product that may be evaluated in this article, or claim that may be made by its manufacturer, is not guaranteed or endorsed by the publisher.
